# Exercise on Prescription: trial protocol and evaluation of outcomes

**DOI:** 10.1186/1472-6963-7-36

**Published:** 2007-03-02

**Authors:** Jes B Sørensen, Jakob Kragstrup, Kirsten Kjær, Lis Puggaard

**Affiliations:** 1 Centre of Applied and Clinical Exercise Sciences, Institute of Sports Science and Clinical Biomechanics, University of Southern Denmark, Campusvej 55, DK-5230 Odense M, Denmark; 2 Research Unit for General Practice, J.B. Winsløws Vej 9A, University of Southern Denmark, DK-5000 Odense C, Denmark

## Abstract

**Background:**

In many countries exercise prescriptions are used in an attempt to initiate a physically active lifestyle in sedentary populations. Previous studies have primarily evaluated low intensive exercise prescription interventions and found moderately positive effects on physical activity and aerobic fitness. In a highly intensive Danish exercise prescription scheme called 'Exercise on Prescription' (EoP) the general practitioners can prescribe EoP to sedentary patients with lifestyle diseases. The aim of this randomized trial is to assess the short- and long-term effects of the EoP scheme. Thus, the aim of this paper is to describe the randomized controlled trial designed for evaluating effectiveness of EoP, and to present results from validations of outcome measures.

**Methods/Design:**

EoP involves a 16-week supervised training intervention and five counselling sessions (health profiles). All patients referred to EoP were eligible for the trial and were offered participation during the baseline health profile. Comparisons between the EoP group and the control group were made at baseline, and after four and ten months. Physiological measures used were maximal oxygen uptake (VO_2_max), glycosylated haemoglobin (HbA1c), bodyweight, and BMI. Patient-reported measures used were physical activity, health-related quality of life, amount and intensity of exercise, compliance with national guidelines for physical activity, and physical fitness. The validation of the cycle ergometer test found a strong correlation between maximal work capacity and VO_2_max, and acceptable test-retest reliability at group level. Calibration of the HbA1c apparatus was stable over ten weeks with minimal use, and test-retest reliability was good. High agreement percents were found for test-retest reliability for the self-administered questionnaire.

**Discussion:**

The trial is designed to provide information about the effectiveness of the EoP scheme. The trial is part of a health technology assessment of EoP, which besides the effectiveness covers the patient perspective, the organization, and the health economy. All three methods validated were found useful for the EoP trial.

## Background

Epidemiological studies have reported a reduced risk of lifestyle diseases and/or mortality with increased level of physical activity [1] and aerobic fitness [2]. A physically active lifestyle is recommended both as part of treatment, prevention and rehabilitation with regard to a number of lifestyle diseases [3]. Despite increasing awareness of the benefits of a physically active lifestyle, large parts of the population remain physically inactive, posing a serious threat to public health [4,5].

In many countries exercise prescriptions are used in an attempt to initiate a physically active lifestyle in sedentary populations. However, a recent population-based analysis questions the impact of exercise prescriptions on increased physical activity at population level [6], and a population-based survey reports that general practitioners (GPs) give physical activity advice and exercise prescription to only 13 % and 3 %, respectively [7]. Furthermore, a recent review concludes that exercise prescriptions only have a moderately positive effect on physical activity level and aerobic fitness [8]. However, exercise prescriptions are still used extensively [9]. Most exercise prescription studies have evaluated GP counselling in combination with low intensive interventions (e.g. possibility to see an exercise specialist [10], pedometers and exercise logs [10], community-based activities and telephone support [11], access to local physical activity facilities and leisure centres [12], telephone booster calls [13], and stage-matched written material [14]). Few studies have evaluated highly intensive exercise prescription interventions [8,15,16], and limited evidence supports the hypothesis that more intensive interventions will lead to larger improvements in physical fitness and physical activity [8,17]. Furthermore, very few studies have used good measures of aerobic fitness and physical activity [18].

In an exercise prescription scheme called 'Exercise on Prescription' (EoP) used in a number of Danish counties GPs can refer sedentary patients with medically controlled lifestyle diseases or risk of developing lifestyle diseases to an EoP programme. EoP is a highly intensive exercise prescription scheme, which is implemented in primary care and includes group-based supervised training and motivational counselling.

The aim of this randomized trial is to assess the short- (four months) and long-term (ten months) effects of the highly intensive EoP scheme on physiological variables (maximal oxygen uptake (VO_2_max), glycosylated hemoglobin (HbA1c, in patients with impaired glucose tolerance), Body Mass Index (BMI), and bodyweight) and patient-reported variables (physical activity, health-related quality of life, amount and intensity of exercise, compliance with national guidelines for physical activity, and physical fitness). Thus, the aim of this paper is to describe the randomized controlled trial designed for evaluating effectiveness of EoP, and to present results from validations of outcome measures.

## Methods/design

The EoP scheme was evaluated in a randomized trial conducted in 2005–2006 with two groups: EoP and control patients. The EoP scheme was launched one year prior to the evaluation and offered by the County of Ribe and the County of Vejle. Patients were referred to the EoP scheme by their GP. GPs act as gatekeepers in the Danish health care system [19].

The motivational counselling and the group-based supervised training intervention in EoP patients were carried out by a physiotherapist in 14 clinics geographically spread throughout the two counties.

### Intervention

**Figure 1:**
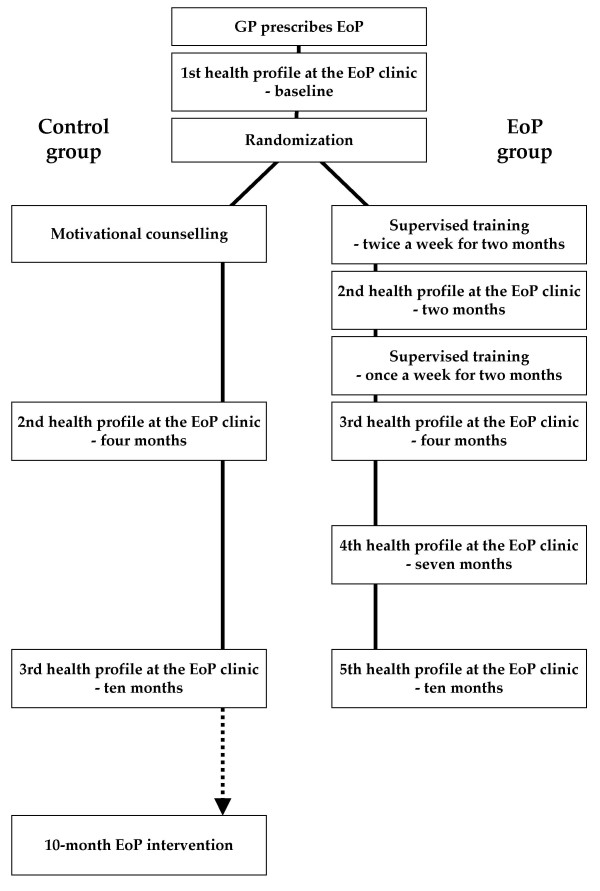
Schematic overview of the randomized trial of 'Exercise on Prescription' (EoP). The general practitioner (GP) prescribes EoP for sedentary patients with medically controlled lifestyle diseases. The patient takes the prescription to an EoP clinic. Patients randomized to the EoP group complete five health profiles and a 4-month supervised training intervention. The health profiles are completed at baseline, and after two, four, seven, and ten months. The supervised training is organized in groups of 8–12 EoP patients, and involves many different forms of physical activity. Patients randomized to the control group receive only the counselling at baseline, and after four and ten months. After taking part in the trial the control group is offered EoP.

Participants randomized to the EoP group followed a supervised group-based training intervention together with 8–12 other EoP patients (including patients not taking part in the trial) (Figure 1). Furthermore, the participants received motivational counselling (health profiles) at baseline and after two, four, seven, and ten months aimed at increasing daily physical activity. The health profiles were also used for making a physical activity plan in collaboration between the physiotherapist and the patient, and the patient was responsible for the execution of the plan. During the first two months two weekly 1-hour training sessions were completed. During the final two months one weekly 1-hour training session was completed, totalling 24 training sessions over four months. The group-based training intervention involved elements of aerobic conditioning (e.g. Nordic walking and aerobics), light strength conditioning (primarily using light weights and high number of repetitions), stretching, and games. The physiotherapists were instructed in focusing on training improving aerobic capacity (more than 50% of heart rate reserve for a minimum of 20 minutes [20]) in the majority of training sessions.

Participants randomized to the control group were called in for a counselling session following randomization (Figure 1). Furthermore, the control group received similar health profiles as the EoP group, but only after four and ten months. After participating in the evaluation the control group was offered EoP.

### Inclusion and exclusion criteria

General practitioners could refer patients with the following characteristics: 1) medically controlled lifestyle diseases, 2) motivated to change of lifestyle, 3) believed to be able to improve health from an increased physical activity level, and 4) willing to pay 750 DKr. (100 €) for the intervention. In addition to giving the referral the general practitioner informed the patient about the benefits of a physically active lifestyle in general.

### Recruitment and allocation

Referred patients contacted an EoP clinic, and an appointment for the first health profile was scheduled (Figure 1). All patients referred to the EoP scheme were eligible for the trial and were offered participation in the randomized trial during the baseline health profile. Recruitment took place over 14 months in 2005 and 2006.

Informed consent was obtained, and volunteering patients were randomized to either the EoP group or the control group. Randomization was carried out by the first authors by means of concealed envelopes containing the name of the group. Group allocation was then reported by mail to the physiotherapist and the patient. Randomization was stratified according to diagnosis (cardiovascular diseases, metabolic syndrome, type II diabetes, and other diseases) in order to distribute diagnosis groups evenly in the two groups.

### Outcome measures

The EoP physiotherapists carried out both the physiological and patient-reported measures. Changes in physiological measures (VO_2_max, bodyweight, BMI, and HbA1c) and patient-reported measures (physical activity, health-related quality of life, amount and intensity of exercise, compliance with national guidelines for physical activity, and self-reported physical fitness) were assessed at the time of the health profiles (Figure 1).

*Maximal oxygen uptake (VO_2_max) *was measured using a maximal indirect cycle ergometer test modified from Pedersen & Nielsen [21], where VO_2_max was calculated from the maximal work capacity (Wmax). All tests were completed on a mechanically braked cycle ergometer (Ergomedic 874E or 828E, Monark, Varberg, Sweden). Rate of perceived exertion was evaluated using the 6–20 point Borg scale [22]. Heart rate was measured using a telemetric system (Polar Vantage NV, Polar Electro KY, Kempele, Finland). Each workload consisted of three minutes of cycling at 60 rpm. A choice of two protocols was available: 1) an initial workload of 30 watts and increments of 30 watts until exhaustion (classic protocol), 2) an initial workload of 30 watts and increments of 60 watts until the subject rated perceived exertion ≥ 13 (somewhat hard). Thereafter increments of 30 watts were used until exhaustion (progressive protocol). The test was continued until the subject was unable to maintain a pedal frequency of 60 rpm despite verbal encouragement. Workload, maximal heart rate and total time were recorded, and Wmax and VO_2_max were calculated.

*Glycosylated hemoglobin (HbA1c) *was measured in capillary blood using the DCA 2000+ (Bayer Diagnostics Europe Ltd., Dublin, Ireland), and was only assessed in patients with reduced insulin sensitivity. HbA1c is an indicator of mean blood glucose level over the last 8–12 weeks and indirectly a measure of glycemic control [23]. In a comparison between DCA 2000+ and the gold-standard technique in HbA1c assessment the correlation was above 0.9, and the test-retest reliability with the DCA 2000+ was above 95 % [24,25].

*Bodyweight *was measured prior to the cycle ergometer test with patients wearing light clothes and no shoes using a commercial scale.

*Body Mass Index (BMI) *was calculated by dividing bodyweight with height squared.

All patient-reported measures were assessed by means of self-administered questionnaires distributed by the EoP physiotherapist during the health profiles. The questionnaire was self-explanatory, easy to fill in, not too extensive, and consisted of preset response categories.

*Physical activity *was assessed using a short questionnaire, which allowed for conversion to MET values [26].

*Health-related quality of life *was assessed using both the SF-12v2 [27] and EQ-5D [28].

*Amount and intensity of exercise *were assessed by asking 'how much exercise do you do during an average week?', and 'at what intensity do you most often exercise?'

*Compliance with national guidelines for physical activity *was assessed by asking 'how many days during an average week are you physically active more than 30 minutes?'

*Self-reported physical fitness *was assessed by asking 'how do you rate your present physical fitness?', 'how do you rate your present physical fitness compared to four months ago?', and 'how do you rate your present physical fitness compared to people your age?'

The outcome measures are surrogate measures for reduction in risk of morbidity and mortality.

All physiotherapists were trained in the different aspects of the evaluation prior to initiating the randomized trial. A manual described all parts of the evaluation in detail, and each clinic was visited several times before and during the evaluation.

### Sample size

Power calculations were performed for expected changes in VO_2_max. VO_2_max at baseline was estimated to 20 ml O_2_*(min*kg)^-1^, expected improvements were 15%, and expected SD was 3 ml O_2_*(min*kg)^-1^. Alpha was set at 0.05 and a power of 0.9 was chosen. Using these assumptions in the 'sampsi' command in Stata 9.0 the sample size was estimated to 22 in each group.

### Statistical analysis

The changes from 0–4 and 0–10 months within the two groups separately will be assessed using paired t-test or Wilcoxon rank sum test depending on distribution. Furthermore, boxplots will be made for delta values for the changes from 0–4 and 0–10 months.

Comparisons between the EoP group and the control group will be made with changes from 0–4 and 0–10 months. The analysis will be performed as an intention-to-treat and carried out using a regression model incorporating delta values, controlling for baseline values, and adjusted for the effect of the different centres (physiotherapist clinics), further robust variance will be applied. For variables not fulfilling the normal distribution of residuals a Wilcoxon rank sum test will be used on delta values. These analyses will be supplemented with 'per protocol analyses'.

Drop-out analyses will be carried out for 0–4 months and 4–10 months. Completers and non-completers will then be compared for the different baseline variables using boxplots. The effect of drop-out will be compared with differences between the EoP group and the control group.

Statistical significance was set at p < 0.05.

### Approval

Local ethics committee registration number VF 20040121. The Danish Data Protection Agency registration number 2004-41-4692. ClinicalTrials.gov registration number NCT00399997.

### Validation studies

Validation studies were carried out for the cycle ergometer test, the HbA1c method, and the self-administered questionnaire. Detailed information about the validation studies can be found in the appendix.

### Wmax cycle ergometer test validation

Validation was performed for both the calculation of VO_2_max from Wmax (equation study) and the comparison of the two different protocols (protocol study). The protocol study aimed at finding a less time-consuming protocol, and evaluating test-retest reliability.

**Figure 2:**
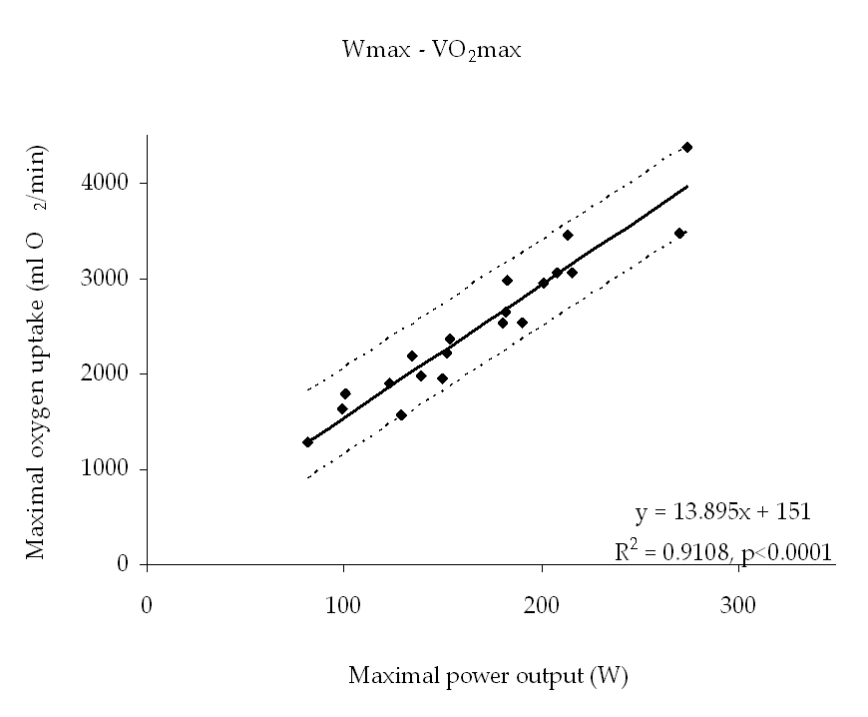
The Wmax-VO_2_max relationship. The maximal work capacity (Wmax) was plotted against the maximal oxygen uptake (VO_2_max), and linear regression was performed. The relationship was highly linear (VO_2_max = 13.895 * Wmax + 151 ml O_2_/min, R^2 ^= 0.9108, p < 0.0001), but prediction intervals were wide (mean ± 1.96 SD, 452 ml O_2_/min).

**Figure 3:**
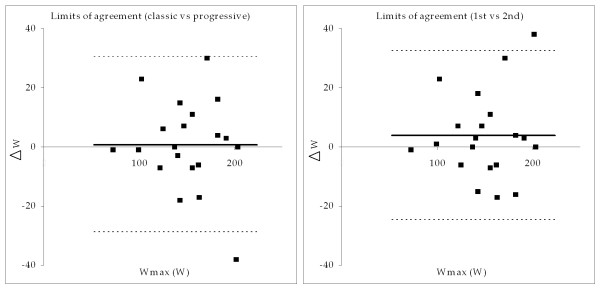
Differential plots with limits of agreement (LOA) for the classic test vs. the progressive (left) test and 1st test vs. 2nd test (right). The individual differences for the two tests were plotted against the individual mean of the two tests. Furthermore, limits of agreement (LOA) were calculated and plotted (dotted lines). LOA was wide in both comparisons (29.6 watt and 28.6 watt, respectively), but no statistical significant differences were found. In both cases coefficient of variation was 6.95%.

A close relationship between Wmax and VO_2_max in a group of EoP patients (Figure 2) and acceptable test-retest reliability at group level (Figure 3, right) were found. Furthermore, results from the two different protocols were comparable at group level (Figure 3, left). However, at individual level the Wmax test was less reliable.

### DCA 2000+ glycosylated haemoglobin validation

Test-retest reliability for the DCA 2000+ apparatus was assessed in healthy subjects. Furthermore, a stability test of the calibration was performed by checking a DCA 2000+ apparatus with a known standard sample once a week over a period of ten weeks.

Good test-retest reliability and low LOA over a period of one week were found. Furthermore, calibration was stable for a period of ten weeks with minimal use.

### Self-administered questionnaire validation

Test-retest reliability for the self-administered questionnaire was assessed for 16 EoP patients, who answered the questionnaire twice within 1–2 weeks. Agreement between the two completed questionnaires was assed using kappa statistics.

The test-retest reliability of the self-administered questionnaire proved good in terms of agreement percent despite low kappa values.

## Discussion

The EoP trial is designed to provide information about the effectiveness of the scheme. The trial is part of a health technology assessment of EoP, which besides the effectiveness covers the patient perspective, the organization, and the health economy [29].

The validation of the Wmax cycle ergometer test demonstrated a close relationship between Wmax and VO_2_max in a group of EoP patients (Figure 2) and acceptable test-retest reliability at group level (Figure 3, right). Furthermore, results from the classic and the progressive protocol were comparable at group level (Figure 3, left). However, at individual level the Wmax test was less reliable since LOA between the classic and the progressive test and between 1st and 2nd test were rather large. The validation of the DCA 2000+ apparatus for measuring HbA1c resulted in good test-retest reliability and low LOA in healthy subjects over a period of one week. Furthermore, calibration was stable for a period of ten weeks. The test-retest reliability of the self-administered questionnaire proved good in terms of agreement percent despite low kappa values. All three methods were found useful for the EoP trial.

## Competing interests

The author(s) declare that they have no competing interests.

## Authors' contributions

JBS drafted the manuscript. JBS and KK designed and completed the validation studies. LP obtained funds for the project. JBS, JK and LP developed the project. All authors read, commented, and approved the final version of the manuscript.

## Appendix

### Wmax cycle ergometer test validation

Subjects were 45 EoP patients (Table 1, top). In the equation study the test was accepted as a maximal effort if blood lactate exceeded 8.0 mM and/or respiratory-exchange-ratio exceeded 1.0. In the protocol study the test was accepted as maximal effort if maximal heart rate exceeded 85% of age-predicted maximum (220 minus age) (Table 1, bottom). Five subjects did not fulfil the criteria and were excluded (three from the equation study and two from the protocol study), and 40 subjects were used for the analyses (Table 1, top).

**Table 1:** Cycle ergometer test validation subjects and results. Top: anthropometric data for the subjects. Bottom: results from the maximal test. Data are presented as mean ± SD and range.

	**Equation study**			**Protocol study**		
	Women (N = 10)	Men (N = 10)	All (N = 20)	Women (N = 16)	Men (N = 4)	All (N = 20)
Age (years)	52 ± 12	52 ± 12	52 ± 11	57 ± 10	53 ± 9	56 ± 10
	34–69	26–66	26–69	39–75	40–60	39–75
Weight (kg)	86.9 ± 16.6	99.0 ± 17.0	93.0 ± 17.5	81.9 ± 14.8	97.4 ± 9.3	85.1 ± 14.9
	61–105	72–129	61–129	62.9–107.0	88.4–108.7	62.9–108.7
Height (cm)	164 ± 8	178 ± 6	171 ± 10	166 ± 7	178 ± 4	168 ± 8
	149–175	172–192	149–192	155–179	174–183	155–183
BMI (kg*m^-2^)	31.9 ± 4.0	31.1 ± 4.7	31.5 ± 4.3	29.8 ± 4.9	30.7 ± 2.2	30.0 ± 4.3
	25.1–37.8	24.2–39.7	24.2–39.7	22.3–36.8	29.2–33.9	22.3–36.8

Heart rate (beats*min^-1^)	160 ± 15	164 ± 13	162 ± 14	160 ± 16	166 ± 17	160 ± 14
	127–179	147–184	127–184	139–185	151–188	139–188
Rate of perceived exertion	18.8 ± 1.2	18.6 ± 1.1	18.7 ± 1.1	18.4 ± 1.1	18.5 ± 1.3	18.5 ± 1.2
	17–20	17–20	17–20	16–19	17–20	16–20
Wmax (W)	145.3 ± 36.6	192.7 ± 57.2	169.0 ± 52.7	142.0 ± 36.0	179.3 ± 22.8	149.9 ± 34.6
	99.2–215.8	81.7–274.3	81.7–274.3	73–202	156–203	73–203
VO_2_max (ml O_2_*min^-1^)	2,102 ± 455	2,896 ± 827	2,499 ± 767			
	1,574–3,065	1,285–4,378	1,285–4,378			
Respiratory exchange ratio (VCO_2_max/VO_2_max)	1.10 ± 0.04	1.06 ± 0.05	1.08 ± 0.05			
	1.05–1.19	0.99–1.13	0.99–1.19			

All tests were completed on a mechanically braked cycle ergometer (Ergomedic 874E or 828E, Monark, Varberg, Sweden). In the equation study pulmonary gas exchange was measured breath-by-breath and averaged over the last 30 seconds to determine VO_2_max (Oxycon Pro, Erich Jaeger GmbH, Hoechberg, Germany). Blood samples were collected from a fingertip and analyzed for blood lactate (YSI 1500 Sport Lactate Analyzer, Yellow Springs Instruments, OH, USA). Rate of perceived exertion was evaluated using the 6–20 point Borg scale [22]. Heart rate was measured using a telemetric system (Polar Vantage NV, Polar Electro KY, Kempele, Finland).

Subjects completed a familiarization test on a separate day prior to the studies. The equation study used the classic protocol. The protocol study compared the classic and the progressive protocols in the protocol study. The classic and progressive protocols were performed in random order and were separated by 3–14 days.

In the equation study linear regression was performed on the VO_2_max-Wmax relationship, and a strong correlation was found (VO_2_max = 13.895 ml O_2_*min^-1 ^* Wmax + 151 ml O_2_*min^-1^, R^2 ^= 0.911, p < 0.0001) (Figure 2). The prediction interval was 452 ml O_2_*min^-1^, which indicated a large individual variability. Some 95% of observations in the population are expected to fall within the prediction interval. The SD used for the prediction interval was the SD of the difference between measured and estimated VO_2_max. In the protocol study the comparison between the classic and progressive test indicated that the protocols result in similar Wmax values at group level (Figure 3, left). Comparison of the 1st and 2nd test found a non-significant increase of 3.9 watt (Figure 3, right). However, the large limits of agreement (LOA) in both comparisons indicated large individual variability [30]. For this reason the Wmax test should primarily be used for comparisons at group level. The coefficient of variation, calculated using the method of Vasikaran [31], was 6.95% in both comparisons.

### DCA 2000+ glycosylated haemoglobin validation

HbA1c was assessed three times over a period of one week in nine subjects (four women and five men). Blood samples were collected from both capillary and vein. Mean difference of HbA1c between venous and capillary blood samples was 0.004% HbA1c, and LOA was 0.39. Using one-way ANOVA resulted in with-in subject SD for the three time points of 0.13 (LOA 0.26) and 0.16 (LOA 0.32) for venous and capillary blood, respectively. Only small variations in the measurement of the standard sample were observed over a period of ten weeks, and the range of deviation from the standard samples was 0.1 to 0.4% HbA1c.

### Self-administered questionnaire validation

Agreement was for SF-12 67–97% (mean 90%), for EQ-5D 69–100% (mean 89%) and for physical activity level 87–99% (mean 93%). However, low kappa values were found for several questions in SF-12 0.19–0.88 (mean 0.49), EQ-5D 0.25–1.00 (mean 0.66), and physical activity 0.19–0.91 (mean 0.62), due to an unbalanced marginal distribution caused by few or no observations in some response categories and a small number of participants in this part of the study. The paradox of high agreement percent and low kappa value has been described by Feinstein and Cicchetti [32].

## Pre-publication history

The pre-publication history for this paper can be accessed here:


